# Choosing healthier foods in recreational sports settings: a mixed methods investigation of the impact of nudging and an economic incentive

**DOI:** 10.1186/1479-5868-11-6

**Published:** 2014-01-22

**Authors:** Dana Lee Olstad, Laksiri A Goonewardene, Linda J McCargar, Kim D Raine

**Affiliations:** 1 Alberta Institute for Human Nutrition, 2-021D Li Ka Shing Centre, University of Alberta, Edmonton, AB T6G 2E1, Canada; 2 Department of Agricultural, Food and Nutritional Science, 4–10 Agriculture/Forestry Centre, University of Alberta, Edmonton, AB T6G 2P5, Canada; 3 Centre for Health Promotion Studies, University of Alberta, 3-300 ECHA, 11405 87 Ave, Edmonton, AB T6G 1C9, Canada; 4 Alberta Agriculture and Rural Development, Government of Alberta, #307, 7000 113 Street, J.G. O’Donoghue Building, Edmonton, AB T6H 5T6, Canada

**Keywords:** Environmental change, Intervention, Nudge, Economic incentive, Healthy food, Dietary behaviors, Community context, Profit, Behavioral economics, Mixed methods

## Abstract

**Background:**

Nudging is an approach to environmental change that alters social and physical environments to shift behaviors in positive, self-interested directions. Evidence indicates that eating is largely an automatic behavior governed by environmental cues, suggesting that it might be possible to nudge healthier dietary behaviors. This study assessed the comparative and additive efficacy of two nudges and an economic incentive in supporting healthy food purchases by patrons at a recreational swimming pool.

**Methods:**

An initial pre-intervention period was followed by three successive and additive interventions that promoted sales of healthy items through: signage, taste testing, and 30% price reductions; concluding with a return to baseline conditions. Each period was 8 days in length. The primary outcome was the change in the proportion of healthy items sold in the intervention periods relative to pre- and post-intervention in the full sample, and in a subsample of patrons whose purchases were directly observed. Secondary outcomes included change in the caloric value of purchases, change in revenues and gross profits, and qualitative process observations. Data were analyzed using analysis of covariance, chi-square tests and thematic content analysis.

**Results:**

Healthy items represented 41% of sales and were significantly lower than sales of unhealthy items (p < 0.0001). In the full sample, sales of healthy items did not differ across periods, whereas in the subsample, sales of healthy items increased by 30% when a signage + taste testing intervention was implemented (p < 0.01). This increase was maintained when prices of healthy items were reduced by 30%, and when all interventions were removed. When adults were alone they purchased more healthy items compared to when children were present during food purchases (p < 0.001), however parental choices were not substantially better than choices made by children alone.

**Conclusions:**

This study found mixed evidence for the efficacy of nudging in cueing healthier dietary behaviors. Moreover, price reductions appeared ineffectual in this setting. Our findings point to complex, context-specific patterns of effectiveness and suggest that nudging should not supplant the use of other strategies that have proven to promote healthier dietary behaviors.

## Background

Emerging evidence indicates that many health-related decisions are often made very quickly, with little conscious thought [1-4]. Thus, although many individuals express a rational intention to eat healthfully, in practice they more commonly select unhealthy foods for immediate reasons such as taste and convenience. This intention-behavior gap has been described by behavioral economists as the product of two interacting information processing systems in a dual process model [2,4]. The first is a cognitive system that processes information in a rational manner, thoroughly weighing all options and selecting the best one. Traditional individual-level approaches engage this system through providing information. The second processes information in a non-cognitive manner, making decisions quickly, reflexively, and often in response to environmental cues. While some food-related decisions are made in a thoughtful and considered manner, most are made automatically using the second system, in response to environmental stimuli [2]. A dual process model of information processing provides a compelling basis for understanding why, despite an abundance of information and education, dietary behaviors have been resistant to change, highlighting the need to identify and target potent environmental drivers of food intake.

In response, behavioral economists have proposed an environmental approach to behavior change grounded in principles of libertarian paternalism, that alters social and physical environments to shift behaviors in self-interested directions without limiting the available options [5,6]. Nudging, as this strategy is commonly called, is libertarian in the sense that it provides choice, but paternalistic because choices are presented in a manner that favors particular outcomes. Nudging is not new. The food industry has been successfully nudging consumers to purchase its (primarily unhealthy) products for decades. However, nudging is relatively untapped within public health. Wansink and Just are perhaps best known for having successfully nudged the purchase of healthier items through increasing their convenience [7,8], variety [9], and visibility [10]. Others too have successfully nudged children to select healthier foods through verbal prompts [11,12], enhancing aesthetic appeal [12], brand characters [13] and increased variety [12]. Studies in adults suggest that descriptive menu labels [14] and increasing the visibility [15] and convenience [16] of obtaining healthier items are also effective in increasing their sales.

While often statistically significant, the impact of nudging has been quantitatively small in many instances [7,15,17], raising questions regarding efficacy. Nudges may therefore be more potent if implemented in combination, or along with more powerful economic incentives, however such investigations are limited, as noted in a recent systematic review [18]. At present, there is no compelling evidence to suggest that nudging alone can improve population health [4]. Studies are needed to test the efficacy of nudges in a variety of populations and settings, to determine optimal combinations, and to ascertain whether nudging is as powerful as more overt tools such as pricing.

In Canada, recreational facilities are publicly funded sport complexes providing access to affordable physical activities. These facilities have been identified as a community setting with substantial potential to improve public health, but which, by virtue of unhealthy food environments, may be paradoxically contributing to obesity risk [19-21]. Managers are receptive to providing healthier options, but are reluctant to do so because they believe patrons will not buy them [20-25]. Nudging is a potentially powerful means of cueing healthier food selection in this setting, which might allow industry to improve food offerings without losing revenue. A literature search was therefore undertaken for promising nudges likely to be feasible for implementation in this context.

The literature search identified several nudges that met this criteria. The first, taste testing, was judged promising because taste is one of the most important determinants of food selection among both children and adults [26-31]. Many individuals and especially children are, however, neophobic and hesitant to try new, healthful foods [32,33]. Although taste testing has been a component of successful school [12,34-37] and grocery store-based [38-41] multicomponent dietary interventions, the independent impact of providing food samples to nudge selection of healthful products is not clear. Similarly, descriptive menu labels are a simple, low-cost strategy used by industry to enhance the attractiveness of menu items, but were found to be relatively untested for their independent potential to cue healthier dietary choices [14,42]. By contrast, the literature review showed that economic incentives have proven consistently capable of shifting the dietary behaviors of children and adults in desirable directions [43,44], suggesting they might augment the impact of these more subtle nudges.

This study assessed the comparative and additive efficacy of two nudges (1. signage with descriptive menu labels; 2. taste testing) and an economic incentive in supporting healthy food purchases by patrons in a naturalistic recreational sports setting. We hypothesized that sales of healthy items would be significantly greater compared to baseline in all intervention periods, with greater increases when nudges were combined, and when they were used together with a pricing incentive.

## Methods

### Study design

#### Overview

This study used mixed methods to quantify the impact of three environmental interventions on sales of healthy items at an outdoor community pool. An initial pre-intervention control period was followed by three successive and additive environmental interventions including: 1) signage with descriptive menu labels, 2) addition of a taste testing intervention, and 3) addition of a price reduction intervention. Following the third intervention a final post-intervention control period was instituted.

The study was conducted at an urban, municipally-operated outdoor pool adjacent to a recreational facility in the province of Alberta, Canada. The pool was open daily from 11 am-7 pm (weather-permitting) for 4.5 months of the year. Two concessions were present on-site. The first, a municipally-operated concession, sold exclusively pre-packaged items including a variety of candy, ice cream novelties, granola bars, dessert squares, potato chips, sugar sweetened beverages, fruit juice, diet soda and water. The other concession was privately operated (hereafter referred to as the target concession), and offered a larger menu consisting of main dishes (sandwiches and wraps), beverages (water, sugar sweetened beverages, smoothies, slushes) snacks and desserts (a variety of ice cream and fruit-based dishes) prepared primarily on-site. Data for the study were collected from May - September, 2012.

This study was approved by the Human Research Ethics Board at the University of Alberta. Concession and municipal managers provided written, informed consent to provide data for the study.

#### ***Menus***

A menu was designed for the target concession that included a variety of well-liked options. Menu items were classified as healthy/unhealthy according to the Alberta Nutrition Guidelines for Children and Youth’s criteria for “choose most often” (healthy), “choose sometimes” (unhealthy), and “choose least often” (unhealthy) [45]. The final menu consisted of 44.4% healthy items (Table 1) and was used during all phases of the study (ie. control and intervention periods). The menu in the municipally-operated concession was unchanged and contained very few (9.1%) healthy items.

**Table 1:** Nutritional characteristics of the target concession’s menu

	**Healthy**^**a**^	**Unhealthy**^**a**^
**Main dishes**	25%	75%
**Snacks and desserts**	50%	50%
**Beverages**	55.6%	44.4%
**Total**	44.4%	55.5%
**Average caloric content per item**	144 kcals	283 kcals

^a^Healthy items met the definition of “choose most often” in the Alberta Nutrition Guidelines for Children and Youth [45]. Unhealthy items met the definition of “choose sometimes” and “choose least often”.

#### ***Periods***

The intervention took place exclusively in the target concession. No changes to product, pricing or promotion were made in the municipally-operated concession during the study. The interventions were additive and their order was determined based on the goals of the study, which were to compare the relative efficacy of single (signage) and multiple (signage and taste testing) nudges alone or together with price reductions, in increasing sales of healthy items. Thus, single and multiple nudges were implemented first to test their sales-stimulating potential in the absence of an economic incentive. Each control and intervention period was instituted for 8 days.

##### 

**Quality assurance** Food service staff at the target concession received training regarding all study procedures prior to the pre-intervention baseline period. Topics included correct preparation of menu items, accurate keying of customer orders on the cash register, and specific details related to each intervention. Following training, the modified menu was introduced in the target concession and a trial period of 5 days was instituted to allow staff to practice study procedures in advance of data collection. Once the study began, researchers were present continuously within the setting to monitor compliance.

##### 

**Pre-intervention** During the pre-intervention period menu items were displayed on 28 × 43 cm panels containing item names, descriptors, prices and colorful photos. Signage was placed 2–3 feet above ground level so that even very young children could easily see and touch the signs.

##### 

**Signage intervention** We developed and pre-tested new descriptive names for healthy items that would appeal to children (Table 2). To draw attention to the new names the height of the panels advertising healthy items was doubled in size and signs were positioned as close as possible to the cash register. Signage for unhealthy items remained unchanged.

**Table 2:** Healthy menu items with descriptive menu labels and reduced prices

**Original name**	**Descriptive menu label**	**Original price**	**30% off reduced price**
Watermelon slushie	Wacky wundermelon slushie	$3.50 (regular)	$2.45 (regular)
		$2.50 (small)	$1.75 (small)
Watermelon and frozen strawberry slushie	Wonderful waterberry slushie	$3.50 (regular)	$2.45 (regular)
		$2.50 (small)	$1.75 (small)
The coco cabana smoothie	Creamy coco banana smoothie	$3.50 (regular)	$2.45 (regular)
		$2.50 (small)	$1.75 (small)
Very berry smoothie	The purple moo smoothie	$3.50 (regular)	$2.45 (regular)
		$2.50 (small)	$1.75 (small)
Water	Iced spring water	$1.00	$0.70
Fresh fruit	Fruit ninja	$1.00	$0.70
Fresh fruit tray with fruit dipping sauce	Fresh fruit dippers with verry berry dipping sauce	$2.50	$1.75
Frozen banana	Frozen funky monkey	$1.50	$1.05
Fruit kebab with fruit dipping sauce	Funtastic fruit kebab with verry berry dipping sauce	$2.50	$1.75
Grilled banana	Grilled bananarama boat	$1.50	$1.05
Roast chicken sandwich	Decked out chicken sandwich	$4.95	$3.46
Loaded teriyaki chicken wrap	Funky teriyaki chicken wrap	$4.95	$3.46

##### 

**Signage + taste testing intervention** After the signage intervention had been in place for 8 days, a taste testing intervention was added. During this period small samples of healthy items (Table 2) were distributed to pool patrons between 1130 am and 3 pm daily for 8 days.

##### Signage + taste testing + price reduction intervention

After the second intervention had been in place for 8 days, a 30% price reduction on healthy items was added (Table 2). Bright red ‘30% off’ signs were placed on the panels advertising healthy items. Post-discount prices of healthy items were below those of comparable unhealthy items.

##### 

**Post-intervention** Following the intervention periods baseline conditions were re-instituted for 8 days.

### Data collection

The primary outcome was the change in sales of healthy items in the intervention periods relative to pre- and post-intervention in the full sample (ie. all patrons who purchased items at the target concession) and in a subsample of patrons whose purchases were directly observed. Secondary outcomes included change in the caloric value of purchases, change in revenues and gross profits, and qualitative process observations.

#### ***Quantitative outcomes***

##### 

**Sales in the full sample of patrons** Itemized cash register sales data for all items sold were collected from both concessions throughout the study. Data from the municipally-operated concession were used to provide an indication of fluctuations in sales patterns due to the passage of time, and whether the interventions influenced food purchases outside of the target concession. The municipality provided information regarding the number of pool users each day.

##### 

**Revenues and gross profits** The target concession provided costs for purchasing raw ingredients and other supplies (eg. cups, spoons) for menu items. This information was used to calculate the food cost per item. Revenues per item were calculated as the number of items sold multiplied by the price. Gross profits per item were calculated as the difference between revenue and food costs. Labor costs were not included as they were similar for comparable healthy and unhealthy items and preparation times for all menu items were minimal.

##### 

**Caloric content** The caloric content of all items on the target concession’s menu was calculated using information obtained from package labels, manufacturer’s websites and where necessary from the Canadian Nutrient File (version 2010) and Food Processor SQL (version 10.11.0 ESHA Research Inc., Salem, Oregon).

##### 

**Quantitative observations of a subsample of concession patrons** We assessed whether sales of healthy items in each study period differed according to demographic characteristics of patrons. To provide an unbiased estimate of purchases, observers directly observed a subsample of patrons’ purchases (40.7% of all items purchased at the target concession) in an unobtrusive manner. Beginning at lunch time, an observer recorded observations for 5 consecutive hours per day, for 2 days per period on at least one weekday and one weekend day. An extra day of data collection per period was added when sales volumes were not sufficiently high on the 2 scheduled observation days. Observers were stationed within close proximity to the cash register and could visually see all patrons, hear items being ordered, and see what was printed on each meal receipt.

For each individual who made a purchase, observers recorded their best estimation of the purchasers’ age (adult alone, child alone, both present), sex, weight status (non-overweight, overweight/obese, unknown), and items purchased. Observations were recorded on purpose-developed forms that had been pre-tested. Observers used figural silhouettes for adults (9 for men, 9 for women) [46] and children (7 for boys, 7 for girls) [47] used in previous investigations to assist in estimating weight status. When children and adults purchased items in groups, observers did not record sex or weight status as it was not possible to record full details for all group members.

The first author and observer trained the second observer, a senior nutrition student, in data collection procedures. Rates of agreement and kappa statistics for inter-rater reliability for demographic variables were moderate to high, ranging from 64% to 93% and from 0.57 to 0.96, respectively, all with p values < 0.001. The kappa coefficients (0.85) and rates of agreement (83% to 100%) were high for identification of menu items. Agreement was lower for four menu items, as slushes (43%)/smoothies (65%) and waffle cones (22%)/regular ice cream cones (46%) were sometimes confused. These discrepancies did not alter our findings because these items have identical health ratings.

#### ***Qualitative process observations***

Qualitative process observations were collected to provide context for, and explain quantitative observations and sales data. The same two observers recorded process observations so that, through prolonged engagement, they could become intimately familiar with the setting and patrons’ behaviors within the setting. One observer recorded qualitative observations of pool patrons, while the other recorded qualitative observations of business operations. Joint observation sessions between observers were held at least once per period to provide corroboration, sensitize observers to other potentially influential environmental factors, and provide opportunities for critical reflection. Patterns in the data and ways to improve data collection were also discussed.

##### 

**Qualitative observations of pool patrons** A single observer recorded qualitative observations regarding patrons’ dietary and physical activity behaviors for 5 consecutive hours per day for 11 days during the study, with 2–3 observation sessions per period excluding the final post-intervention phase. This observer adhered to principles of passive participation [48] in which she was regularly present in the setting but did not participate to any significant extent in pool-related activities.

##### 

**Qualitative observations of business operations** The first author observed the operation of the business from the perspective of an active participant [48]. The observer immersed herself in the setting, working alongside managers and staff to become familiar with many aspects of the business, including routine tasks such as procurement, food preparation, customer service, promotions and staff management. This hands-on approach provided an in-depth perspective of the practical realities of the industry, and the feasibility of using environmental change strategies in this context. It also ensured fidelity to the study protocol, as the observer could directly monitor delivery of the interventions and data collection procedures.

### Data analysis

#### ***Quantitative outcomes***

An analysis of covariance using the mixed procedure of SAS version 9.2 (Cary, NC) was used to estimate the impact of the interventions on sales in the target concession considered in three ways: 1) number of items sold, 2) the caloric content of items sold, and 3) revenues and gross profits. The main effects considered were period (ie. pre- and post-intervention and the three intervention periods), type of item (ie. main dishes, side items, snacks and desserts) and nutritional quality (ie. healthy, unhealthy), and all interactions. The main effects of type of item and associated interactions are beyond the scope of the current paper and will be discussed in a future publication that examines the types of healthy items that achieved the highest sales. Sales during the pre- and post-intervention periods did not differ significantly and therefore they were combined. The dependent variable means were adjusted for the highest air temperature reached each day (Canadian National Climate Data and Information Archive), hours of operation in the target concession, and the number of pool patrons. The number of adult patrons was the only significant covariate. Inclusion of a term indicating whether sales occurred on a weekend or weekday did not alter estimates, and therefore this term was not included in the final model.

A maximum likelihood analysis using proc catmod and proc freq (SAS version 9.2, Cary, NC) was performed to assess the impact of the interventions on purchases by individuals in the directly observed subsample, with the nutritional quality of menu items modeled as a categorical dependent variable (healthy/unhealthy). The main effects considered were period (pre and post-intervention and the three interventions periods), and purchasers’ age (eg. adult alone, child alone or both present), weight status and sex. Sales differed significantly in the pre- and post-intervention periods and therefore they were kept separate for the analysis. All 2-way interactions were included in all models. Observations where the purchasers’ weight status was uncertain were removed from the data set (n = 139 purchases), as were observations of pregnant women (n = 6 purchases), yielding a final sample of 2512 items sold.

When the results of the primary analyses were significant, post-hoc t-tests and 2 x 2 tables were used to determine which means differed significantly from one another. Statistical significance was indicated at p < 0.05.

#### ***Qualitative process observations***

Qualitative observations were transcribed and analysed using thematic content analysis. Observations were grouped into themes that described similar events, such as parent–child interactions around food, and patrons’ reactions to the various interventions. Comparison of findings from each period showed that patrons’ behaviors were similar across all periods, and thus observations were integrated across periods. Dominant themes were identified based on the frequency of their occurrence, and narratives were constructed summarizing the most common observations pertaining to each theme. Peer-debriefing served to verify the findings.

## Results

### Overall sales at the target concession

During the course of the study there were 6175 items sold in the target concession, of which 40.8% were healthy (Table 3). The number of healthy items sold was significantly lower than the number of unhealthy items sold (p < 0.0001).

**Table 3:** **Mean daily sales and gross profits in the full sample,** n ± SEM (%)

	**Pre- and post- intervention**^**a**^	**Signage**	**Signage + taste**	**Signage + taste + price**	**P value for interaction with period**	**Overall mean for all periods**	**P value for main effect**
***Mean daily number of items sold***^***b***^							
**Dietary quality**					*NS*		*< 0.0001*
Healthy items	18.1 ± 2.4 (37.7)	21.5 ± 3.2 (42.3)	18.4 ± 3.3 (35.7)	27.4 ± 3.2 (46.5)		21.4 ±1.5^*^ (40.8)	
Unhealthy items	29.9 ± 2.4 (62.3)	29.4 ± 3.2 (57.7)	33.1 ± 3.3 (64.3)	31.5 ± 3.2 (53.5)		31.0 ± 1.5 (59.2)	
**Mean total daily sales**	24.0 ± 1.8 (23.0)	25.4 ± 2.3 (24.3)	25.7 ± 2.4 (24.6)	29.4 ± 2.3 (28.1)		25.7 ± 1.8	*NS*
***Mean daily number of calories sold***^***b***^							
**Dietary quality**					*NS*		*< 0.0001*
Healthy items	3067 ± 636 (27.6)	3860 ± 847 (33.0)	2766 ± 877 (23.5)	4955 ± 857 (37.0)		3662 ± 396^*^ (30.5)	
Unhealthy items	8041 ± 636 (72.4)	7821 ± 847 (67.0)	8999 ± 877 (76.5)	8448 ± 857 (63.0)		8327 ± 396 (69.5)	
**Mean total calories sold**	5554 ± 480 (23.2)	5841 ± 606 (24.4)	5883 ± 647 (24.5)	6701 ± 620 (27.9)		5907 ± 381	*NS*
***Mean daily gross profits in dollars***^***b***^							
**Dietary quality**					*NS*		*< 0.0001*
Healthy items	40.52 ± 6.20 (35.0)	46.05 ± 8.25 (38.3)	38.92 ± 8.53 (31.5)	37.04 ± 8.34 (31.8)		40.63 ± 3.85^*^ (34.1)	
Unhealthy items	75.29 ± 6.20 (65.0)	74.27 ± 8.25 (61.7)	84.60 ± 8.53 (68.5)	79.49 ± 8.34 (68.2)		78.41 ± 3.85 (65.9)	
**Mean total daily gross profits**	57.91 ± 4.67 (24.3)	60.16 ± 5.91 (25.3)	61.76 ± 6.29 (25.9)	58.26 ± 6.03 (24.5)		59.20 ± 3.82	*NS*

n = 6175 items sold during the study; ANCOVA was used to test for main effects and interactions.

^a^Values for the pre- and post-intervention periods were combined as they were not significantly different.

^b^Values are adjusted for daily hours of operation, number of pool patrons, and maximum temperature reached.

^*^Value differs significantly from the corresponding value for unhealthy items, p < 0.05.

### Overall revenues and gross profits at the target concession

**Figure 1:**
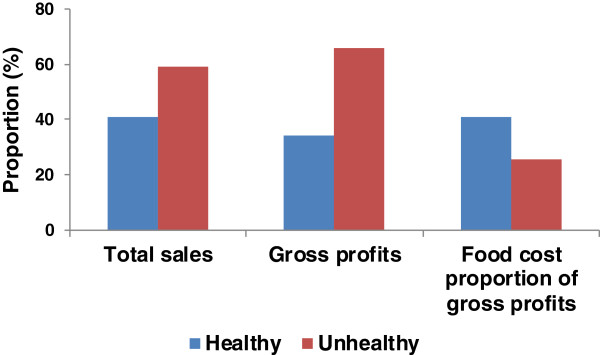
**Total sales, gross profits, and food costs as a proportion of gross profits for healthy and unhealthy items across the five study periods in the target concession.** Total sales represents the proportion of items sold that were healthy and unhealthy across all periods. The share of gross profits from healthy and unhealthy items represents the difference between total revenues and total food costs (cost of purchasing raw ingredients) for healthy and unhealthy items, respectively, divided by total gross profits for all items. Food cost as a proportion of gross profits was calculated by dividing total food costs (cost of purchasing raw ingredients) for healthy and unhealthy items by gross profits for healthy and unhealthy items, respectively.

Average daily revenues (data not shown) and gross profits (Table 3) from unhealthy items were significantly greater than from healthy items (p < 0.0001), with healthy items generating 34.1% of gross profits (Figure 1). The mean daily food cost as a proportion of gross profits was higher for healthy compared to unhealthy items (Figure 1).

The cost to implement the signage intervention was approximately $1500, while the cost to add taste testing approached $200. The additional cost of the price reductions was nearly $600 in lost revenue.

### Impact of the interventions at the target concession

Total sales volumes, the number of calories purchased, and revenues and gross profits from healthy and unhealthy items did not differ by period in the target concession (Table 3).

### Impact of the interventions at the municipally-operated concession

Total sales volumes and sales of healthy and unhealthy items did not differ by period in the municipally-operated concession.

### Demographic characteristics of patrons in the subsample and association with food purchases

Observers witnessed the purchase of 2512 items at the target concession (40.7% of all items sold at the target concession); 41.1% of these items were purchased by adults alone (n = 650 adults observed, 64.0% female, 38.7% overweight/obese), 15.9% by children alone (n = 342 children observed, 55.8% female, 14.3% overweight/obese) and 43.0% by adults and children together (n = 449 groups observed).

More than 41% of items purchased by individuals in the subsample were healthy. The proportion of healthy items sold differed according to who was present during the purchase (p < 0.01; Table 4). When only adults were present, 43.5% of items purchased were healthy, significantly more than when both adults and children (39.0%), or only children (35.8%) were present. These trends were similar when the caloric value of purchases was examined. When only children were present the caloric value of purchases was significantly higher (260 ± 11 kcals) than when adults alone (225 ± 5 kcals) or both children and adults (212 ± 5 kcals) were present (p < 0.001).

**Table 4:** Proportion (%) of purchases that were healthy in each period in the subsample according to purchaser and characteristics of the purchaser

	**Pre-intervention**	**Signage**	**Signage + taste**	**Signage + taste + price**	**Post-intervention**	**P value for interaction with period**	**Overall mean for all periods**	**P value for main effect**
**Purchaser**						*NS*		*0.0068*
Adult only	39.8	40.8	42.4	45.3	46.8		43.5^†^	
Child only	26.6	27.3	47.5	35.8	40.8		35.8^‡^	
Both present	30.6	35.4	40.3	46.8	35.6		39.0^‡^	
**Weight status**^**a**^						*0.0014*		*0.0125*
Non-overweight	32.6	44.7^†^	46.1	40.5^†^	45.9		42.1	
Overweight	35.7	27.5^‡^	35.6	50.8^‡^	43.4		39.7	
**Sex**^**a**^						*0.0094*		*NS*
Male	30.3	38.6	33.6^†^	50.3^†^	42.5		41.3	
Female	35.3	37.5	50.6^‡^	38.0^‡^	46.0		41.3	
**Overall mean**	33.7	37.9	43.9^*^	43.3^*^	44.9^*^		41.3	*0.0048*

n = 1032 items purchased by adults alone, n = 400 by children alone, and n = 1080 by adults and children together; A chi-square analysis was used to test for main effects and interactions.

^a^The weight status and sex of the purchaser were recorded for transactions involving adults only and children only. They were not recorded when both adults and children were present during the transaction.

*Significantly different from pre-intervention, p < 0.05.

^†,‡^Values within a column with different superscripts are significantly different for that effect, p < 0.05.

**Figure 2:**
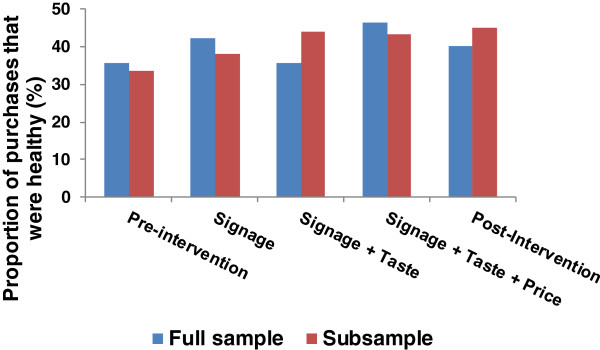
**Purchase of healthy items in the full sample and in the subsample during each of the five study periods.** The full sample includes purchases made by all patrons in the target concession in each study period. Values are derived from cash register sales data. The subsample includes purchases made by patrons whose food purchases were directly observed (40.7% of all items purchased in the target concession).

The proportion of healthy items sold differed by period in this subsample of patrons (p < 0.01; Table 4). An initial 12.7% increase in sales of healthy items during the signage intervention did not reach statistical significance, although the signage + taste testing and the signage + taste testing + price reduction interventions increased selection of healthy items relative to the pre-intervention period by 30.4% and 28.7%, respectively. These increases were maintained in the final post-intervention period, as sales of healthy items remained 33.3% above pre-intervention levels. Sales of healthy items were equivalent across all three intervention periods and the post-intervention phase. For purchases where only adults or only children were present, the effectiveness of the interventions differed according to the weight status and sex of the purchaser, with overweight/obese individuals exhibiting greater sensitivity to the signage + taste testing + pricing intervention and less to the signage intervention compared to those who were not overweight, and males being less responsive to signage + taste testing but more responsive to the signage + taste testing + pricing intervention compared to females (p < 0.01). Figure 2 compares findings by period for the full sample and the subsample.

### Qualitative observations of patrons

Patrons were primarily children accompanied by their parents, and most often by their mothers. Many races were represented, however patrons were primarily Caucasian. There was a sense of enthusiasm and fun in the atmosphere, with much laughter and play. During their visit, patrons alternated between time spent in and outside the pool. Almost all patrons ate at some point during their visit, and most ate intermittently. It was common for families to pack a picnic lunch and supplement with items from the concessions. Social influences were evident around food. Children who approached the target concession with their peers often purchased identical items. Parents also exerted significant control over their children’s eating. They determined the range of choices on offer and were often the ones to initiate eating. Many were observed prodding their children to eat now, to wait to eat until later, to eat only certain items, or to eat at all. Children, however, were also accorded substantial input into food-related decisions as evidenced by the parental-child discussions that occurred around food.

During the interventions children were observed interacting with the colorful displays, however few ordered the fresh fruit they were promoting. During taste testing most patrons were enthusiastic to sample items, although a small minority declined to take a sample. Few patrons commented on the price reductions. Overall, very few patrons inquired about the healthfulness of menu items.

### Qualitative observations of business operations

Challenges associated with offering healthy items in a commercial setting are detailed below.

#### ***Complying with provincial nutrition standards***

While it was relatively simple to create healthy side items for the target concession’s menu (eg. fresh fruit trays), it was much more difficult to develop healthy main dish items that met the provincial nutrition standard for “choose most often” using commercially available ingredients, as for instance, items with ≥ 300 kcals could not exceed 700 mg of sodium. Some items could therefore not be made to fit the “choose most often” standard.

#### ***Food preparation***

It was challenging to ensure consistent preparation of healthy menu items in a busy environment with rotating staff. Even slight deviations, such as adding an extra slice of chicken breast to sandwiches, using a regular versus a low sodium sauce, or preparing a wrap on a white rather than a whole wheat tortilla could cause a healthy item to be classified as unhealthy. Precise control of portion sizes was not possible due to time constraints, as staff did not have time to measure individual ingredients.

#### ***Patron requests***

Patrons sometimes requested unhealthy modifications to healthy menu items, such as adding chocolate sauce or whipped cream to fruit. Although infrequent, managers felt compelled to accede to such requests.

#### ***Communication***

It was challenging to market healthy items in a manner that communicated their healthfulness without stigmatizing them as inferior in taste [49]. Signage had to be understandable and usable within the few seconds typically allocated to food selection in restaurant settings while using limited text.

## Discussion

To successfully navigate the obesogenic food landscape requires constant vigilance, a task that is cognitively depleting and therefore difficult to perform consistently [2,50]. By rearranging the food environment in a manner that facilitates healthier choices, nudging might help to counter the environmental push to select unhealthy options. This study found mixed evidence for the efficacy of three approaches to nudging healthy dietary choices at a population level. Overall, single or multiple nudges, or multiple nudges concurrent with price reductions did not influence the sale of healthy items at a community pool. Direct observations of a subset of patrons’ purchases, however, showed an approximately 30% increase in sales of healthy items when a signage + taste testing intervention was implemented, an increase that was maintained when prices of healthy items were reduced by 30%, and even when all interventions were removed.

It is unclear why results differed in the full and subsamples. The subsample captured a large proportion of total purchases during the study (40.7%), albeit still a minority. As previously described, inclusion in the subsample was determined exclusively by the time of day purchases were made, and coincided with the busiest times of day. Patrons who purchased items during these times may therefore have differed from those who purchased items at other times in a manner that made them more responsive to changes in the food environment. All observations included the lunch time period, and thus it is possible that patrons may have been selecting a meal or a supplement to a meal, as opposed to a treat or a snack. The proportion of healthy items purchased by individuals in the full (40.8%) and subsample (41.3%) was not different, however. It is also possible that item misclassification on the part of observers might account for our mixed findings. This appears unlikely, however, because there was high congruence between observers and they could visually see all items being ordered and the printed list of items on receipts. It is also possible that patrons ordered healthy items because observers were present, although this too appears unlikely as observers were present during both control and intervention periods and their presence and purpose were not readily apparent. Differences in the statistical procedures used to analyse the two data sets might also be responsible, although our methods are consistent with others [51].

### Intervention impact

We implemented two nudges that have received little study, finding mixed evidence for their efficacy. We first tested the efficacy of descriptive menu labels, a strategy commonly used by the restaurant industry to improve consumers’ taste expectations [42]. Wansink et al. [14] previously showed that sales of targeted (healthy and unhealthy) items in a University faculty cafeteria increased by 27% when they were given descriptive menu labels. We created descriptive menu labels for healthy items only, and although we increased the size of the signs on which they were placed, there was no impact of this change on sales of healthy items. When a taste testing intervention was added the results were mixed, as there was no apparent impact in the full sample, however sales in the subsample increased by 30% relative to baseline. The latter findings are in line with studies showing that repeated exposure to healthier foods can counter children’s naturally neophobic tendencies [33], and that free product samples distributed to neophobic young adults can increase selection of unfamiliar healthful food products by 14.6% [32]. Our findings in the subsample suggest an even stronger impact of taste testing, as participants were in a natural setting where they were required to pay for a full portion of the healthy items they had tasted. Qualitative studies suggest that children are particularly reluctant to expend money on new food choices or on fruit and vegetables which might taste bad, as opposed to packaged junk foods which always taste the same [30]. Our findings suggest taste testing might help to reduce this perceived risk, thereby nudging purchase of novel, healthier items in some community settings.

Systematic reviews have found subsidies/price reductions on healthier foods to be an effective means of increasing their purchase and consumption in a variety of settings [44,52,53]. This has not always proven to be the case in single [54,55], or combined interventions [44,52], however, suggesting that price may not always drive food purchases and that some populations are more price sensitive than others. In particular, low income populations, for whom food represents a larger proportion of total expenditures, are predictably more price sensitive [56-60].

In the present study, sales of healthy items remained constant in the full and subsamples when a pricing intervention was added, suggesting that price reductions did not incent purchase of healthy items in this context. Although it is possible that the impact of the signage + taste testing intervention in the subsample might have waned had a price reduction not been added, it appears more probable that price reductions were ineffective because our study sample likely represented a population with higher socioeconomic status (SES). There are several reasons to suspect that pool patrons represented a higher SES group. The study took place in one of the wealthiest jurisdictions in North America [61,62] and the pool was proximal to several wealthy neighborhoods. In addition, the pool was not readily accessible on foot or via public transit, and had a relatively high entrance fee, factors that have been shown to deter youth in low SES groups from participating in physical activity [63]. Observers also noted that many families appeared well off, paid cash for their purchases, and that few children were overweight/obese. Higher SES populations might not perceive a 30% financial savings to be worth the non-monetary costs (poorer taste, reduced satisfaction) of consuming healthy items. Alternatively, price reductions might have been more effective had other healthy items been targeted, as the efficacy of price reductions differs by item [54,64]. Many of the healthy items targeted in this study contained fruit, and the demand for fruit is relatively inelastic [56,57,65,66]. Finally, given that the final price of discounted items was not posted (ie. signage placed on healthy items indicated that they were 30% off), it is possible that children or others with limited numerical skills did not understand the potential savings to be had.

Although the addition of a pricing intervention did not appear to influence purchase of healthy items overall, individuals in the subsample who were overweight/obese exhibited a greater sensitivity to the addition of a pricing intervention relative to individuals who were normal weight. It is possible that the greater sensitivity of overweight/obese persons to the signage + taste testing + price reduction intervention may reflect heightened price sensitivity due to their relatively lower SES, as individuals of lower SES tend to have higher body weights compared to those of higher SES [67,68]. These results contrast with those of Epstein et al. [69], who observed that obese mothers were less price sensitive than their nonobese counterparts. Males in the subsample also exhibited greater sensitivity to the addition of a pricing intervention relative to females. It is not clear why this was the case. Moderators of price sensitivity have rarely been examined, and gender-specific impacts of pricing interventions were not reported in a recent systematic review [52]. Subgroup-specific findings should be interpreted with caution, however, in light of discrepancies in findings between the full and subsamples, and the moderate kappa coefficients for inter-rater reliability on demographic variables. Our findings in this respect highlight the need to consider effect modification in future studies.

In the full sample sales of healthy items did not differ in the pre- and post-intervention phases, however in the subsample sales of healthy items remained approximately 30% above baseline values in the post-intervention period. This result may indicate that patrons in the subsample who increased their purchase of healthier items in the signage + taste testing and signage + taste testing + price reduction phases learned to prefer the healthier menu items, and therefore continued to purchase them when all the interventions were removed. Alternatively, if the same patrons did not frequent the pool on a weekly basis the finding that sales of healthy items remained 30% above baseline values in the post-intervention phase might indicate that something other than the interventions, such as differences in the clientele or an unobserved change at the pool, was responsible for the ~30% increase in sales of healthier items observed. A marked shift in the clientele coinciding with the start of the signage + taste testing intervention appears unlikely, however, and observers were almost continuously present at the pool and did not observe any changes other than those implemented as part of the intervention. Thus, the most plausible explanation is that the same patrons returned to the pool on a weekly basis and findings represent a true impact of the interventions on their food purchases.

Limited impacts of the interventions are perhaps unsurprising in light of the social ecologic framework, which suggests that health behaviors are shaped by reciprocal interactions among individual, social, and environmental factors. Nudging is a very subtle technique, perhaps too subtle to counter the powerful influence of other environmental factors, such as food marketing, or individual factors such as food preferences or purchasing intentions. Indeed, the impact of nudging on food selection in many studies has been relatively small [7,15,17] and inconsistent. Nudges that have proven effective in one context [15,70,71] have had null [51,72], or even opposite impacts in others [73], and outcomes sometimes differ widely for individual items [15,70,71,74]. Our findings are similar, as nudges that were not effective in the full sample were effective among a subsample of patrons, and their impact differed significantly according to the weight status and sex of the purchaser, suggesting differential sensitivity to specific food environment characteristics. Nudges might be more effective if incorporated within multicomponent interventions, or if carefully matched to the particular circumstances of a target population and setting.

Other explanations for our findings might include the fact that many healthy menu items were similar to the contents of patrons’ home-packed lunches, making them less attractive compared to many unhealthy menu items which could not be brought from home due to temperature restrictions (eg. ice cream cones, grilled cheese sandwiches). Second, given that few children were overweight, parents may not have perceived a need to closely regulate children’s intake of unhealthy items [75]. Third, the interventions may have had limited reach. Although the signage advertising the new names and price reductions on healthy items was colorful and prominent, it may not have captured the attention of consumers in the few seconds typically allocated to food selection in away-from-home settings [51,75-77], particularly given the excited atmosphere [78]. Similarly, not all patrons participated in taste testing. Fourth, we only promoted the sale of the most healthy items on the menu. Other studies have combined healthy and moderately healthy items into a “healthier” category. Our results may have differed had we also promoted the sale of moderately healthy items, as the taste profiles of these foods are more compatible with consumer taste preferences. Lytle [79] has suggested that when food access is limited by factors such as low-income, individuals may be more susceptible to influences within the physical food environment. Thus, it is possible that the higher SES of the study sample might also underlie their relative insensitivity to the interventions. Finally, health is only one of many things that individuals value. Children, in particular, have difficulty perceiving the long-term health consequences of dietary choices, and tend to prioritize taste, particularly when eating outside the home [30,80]. Visits to the pool were a fun family outing and therefore parents may have been more likely to allow indulgences and to accede to children’s food requests [81,82].

### Other findings

Compared to purchases made by adults alone and/or by children and adults together, when children were alone they purchased more unhealthy items and items with significantly more calories. Children perceive that purchase and preparation of fruits and vegetables are adult tasks [30]. Thus, it may be wise for parents to at least accompany children during food selection. Notably, however, adult choices were not substantially better than the choices made by children alone, a finding also observed by others [80,83,84]. In qualitative studies parents admit that they purchase unhealthy foods for their children because other concerns, such as convenience and cost sometimes take precedence over health [85-87]. Indeed, adults may be equally susceptible to environmentally-cued food selection, suggesting that all groups may benefit from increased availability of healthy options in recreational sports settings. It is important that adults select healthier options not only for their children, but also for themselves, to support health, and because parental role modeling significantly influences the dietary behaviors of children [88,89].

In contrast to industry’s contention that healthy items do not sell in recreational sports settings [24,25], healthy items were popular among pool patrons and represented 40.8% of items sold. Their share of gross profits was somewhat lower, at 34.1%, as the cost to purchase raw ingredients was higher for healthy foods relative to the profit they generated. Managers can find ways to further minimize food costs, however, as minimizing food costs was not an explicit study goal. In addition, lower profits on healthy items could be offset by increasing the price of unhealthy items [58,90]. None of the interventions increased overall sales volumes as has been seen in other studies [51,91,92], a beneficial finding from a public health perspective, but one that is contrary to the profit motive of industry; however, neither did they adversely affect gross profits, and all were relatively inexpensive to implement and administer. This study also identified a number of non-monetary challenges related to offering healthy items that were encountered by industry. The importance of working with the food industry to improve food environments has been recently highlighted [21,93] and it will therefore be important to address these barriers to ensure they do not impede much needed improvements to food environments.

### Strengths and limitations

Researchers implemented all interventions in conjunction with concession staff and monitored them closely to ensure high fidelity. Thus, null results cannot be attributed to poor execution of interventions. The study was performed in a real-world setting with all of its constraints and supports, increasing the validity of findings. An important strength of this study was that anonymous sales data were augmented by objective measures of food selection in a subsample of patrons for whom selected demographic characteristics were recorded. These strengths are balanced by several limitations, as observer error in this respect may have introduced bias. Our findings related to sex and weight status-specific effects of the interventions apply only to purchases made by adults and children alone, and should therefore be regarded as a preliminary indication of the need for additional study of effect modifiers in this context. We collected observations of patrons who purchased items in the target concession, however these individuals do not necessarily represent those who contributed to the food purchasing decision or those who consumed the items. It is also not clear whether our findings have implications for dietary intake and body weight outcomes. If the changes observed are contextually specific, are not sustained over time, or do not lead to displacement of energy-dense foods in the diet, then these interventions may have little to no real impact. Given that the interventions were additive it was not possible to isolate their individual effects. Moreover, findings may not be generalizable to other settings and populations, or to sales of other healthy foods.

### Future directions

There is no precise, operational definition of nudging [4]. To date, nudging has principally been used in an ad hoc manner and there is a need for a more robust theoretical underpinning to inform development and implementation of interventions [94]. A variety of data will be needed to achieve this outcome. Future studies should compare the relative efficacy of nudges implemented in different populations and settings, alone or in combination, and at multiple decision points such as when selecting a restaurant, at the point of ordering, during meal consumption, and at subsequent meals. The current literature suffers from heterogeneity in study outcomes, intervention sites, types of interventions, participants, outcome measures and types of meals [18], and it will therefore be important that future studies be designed in a manner that facilitates cross-study comparisons. Studies should also incorporate process measures to assist in understanding why some nudges work in some settings and others do not. Longer-term studies are needed, as the efficacy of nudges implemented in the same manner for the same foods might wane over time.

## Conclusions

The notion that food choices can ever be free and independent is illusory at best, as the environment must always be arranged so as to influence choice in some manner [6]. Nudging’s soft paternalistic approach may represent an acceptable compromise between libertarians who advocate for free choice and those wishing to eradicate negative environmental exposures. This study, however, found mixed evidence for the efficacy of nudging in cueing healthier dietary behaviors. Moreover, price reductions appeared ineffectual in this setting. Our findings point to complex, context-specific patterns of effectiveness. Given nudging’s small and inconsistent impacts to date, it should not supplant the use of other proven strategies, but should be regarded as one more tool in the obesity prevention toolbox that may be useful in particular contexts. The challenge for public health will be to identify optimal combinations and contexts in which to apply nudges and leverage their strengths within a social ecological framework. Premature reliance on nudging in the absence of such information could prove harmful if more effective interventions are neglected as a result [18].

## Abbreviations

ANCOVA: Analysis of covariance; Kcals: Kilocalories; SEM: Standard error of the mean; SES: Socioeconomic status.

## Competing interests

The authors declare that they have no competing interests.

## Authors’ contributions

DLO: designed the study, collected, analysed and interpreted the data, wrote the manuscript, obtained funding; LAG: analysed and interpreted the data, edited the manuscript; LJM: designed the study, interpreted the data, edited the manuscript; KDR: designed the study, interpreted the data, edited the manuscript. All authors read and approved the final manuscript.
